# Editor's choice: Infections, sexual reproductive health and non-communicable diseases

**DOI:** 10.4314/ahs.v24i4.1

**Published:** 2024-12

**Authors:** James K Tumwine

We bring you this December 2024 Issue of *African Health Sciences* in a season when the Africa region is grappling with infectious diseases such as Mpox. Fortunately, the Marburg outbreak in the region has been nipped in the bud, and there is a semblance of relief in East Africa. In this issue, we bring you articles in 4 main categories: Infectious diseases; sexual and reproductive health; Non-communicable Diseases; Child Health, and other issues

## Infectious Diseases

In this section, we bring you a paper on genomic variations of Mycobacterium tuberculosis in Uganda^1^, oncogenic human papilloma virus in Ethiopia^2^, and molecular epidemiology of hepatitis C in Pakistan^3^.

There are two extra papers on hepatitis B and C^4^ and the other on hepatitis B and E^5^. The next one is on the diagnosis of microfilaria^6^, Leishmaniasis^7^, and Schistosomiasis^8^.

Not to be outdone, COVID-19 resurfaces - first on a treatise on wound infection^9^, and effects of the vaccine^10^.

The infections section is concluded with surgical site^11^ and orthopedic^12^ infections. A seminal case report on toxoplasmosis in a patient with cancer^13^ is the icing on the cake.

## Sexual Reproductive Health

This thrusts us into a diverse but very interesting section on sexual reproductive health issues. First, we have a paper on a randomized control trial for hysterectomy^14^, followed by two studies on pre- and post-menopausal women. One reports on adnexal masses in premenopausal women^15^, followed by endometrial thickness in post menopause^16^.

Next, we have papers on fertility outcome of intravaginal insemination among women with unconsummated marriages^17^, followed by surrogate maternity in Eastern Europe^18^.

Sexual behaviour and condom use among older adults living with HIV/AIDS^19^ is intriguing as are renal complications of uterine fibroids^20^ in Nigeria. Prevention of obstetric fistula^21^, mHealth for boosting antenatal care [ANC]^22^ and use of herbal medicine^23^ among ANC attendees follow.

We then delve into the perinatal period with papers on detection of fetal heart diversity among patients with pregnancy-induced hypertension 24, second trimester pregnancy loss^25^, and Caesarean sections among East African refugees^26^.

The section ends with a trans-cultural tool for detection of postnatal mental illness^27^.

## Non-communicable diseases (NCDs)

This launches one into the section on NCDs. It consists of papers on prognostic serum anti-inflammatory markers for breast cancer^28^, adenocarcinoma of the appendix^29^, and anti-inflammatory effects of Punica granatum L. on leukemic cell lines^30^.

There are two cancer papers including one on the effect of radio-frequency ablation on ground glass nodules^31^, and a case of schwannoma of the nasal septum^32^.

Quite naturally, stroke papers follow and here we have one on the efficacy of acupuncture on stroke^33^, while the other is on the treatment of stroke complications^34^. Then come orthopaedic/surgery papers including one on fibrosis of the intervertebral disc^35^, rheumatoid arthritis^36^, anesthesia for total hip arthroplasty^37^, osteoporosis in rheumatoid arthritis^38^, and treatment of gout^39^. Then we have a paper on the role of ginsenoside in CNS disease^40^, physiotherapy for Parkinson's disease^41^ and uptake of refractive error services^42^.

The next subsection has found a temporary home under NCDs and these include irritable bowel syndrome^43^, male adolescent smoking^44^, pesticide exposure among farmers in Palestine^45^, treatment for periodontitis^46^, mobile devices for drug education^47^, and diabetic nephropathy^48^.

The final section is on Child Health issues. These include effectiveness of a breastfeeding program^49^, malnutrition of children of adolescent mothers^50^, stunting among children^51^, and exclusive breastfeeding in Nigeria^52^. A seminal paper of hearing loss among school children in Ethiopia brings us to the end of this Editor's choice for this December 2024 issue of African Health Sciences.

We thank our colleagues and partners for giving us the opportunity to publish high quality and truly open-access papers of relevance to Health sciences in Africa and the diaspora. We wish you a very enjoyable festive season and a happy 2025.
